# Detection of Viable *Nannizziopsis guarroi* in Housing Environments Prior to Dermatological Lesion Development in Bearded Dragons (*Pogona vitticeps*) †

**DOI:** 10.3390/ani16020275

**Published:** 2026-01-16

**Authors:** Jacob P. Dalen, Amanda D. Wong, Laura Adamovicz, Nicholas C. Liszka, Krista A. Keller

**Affiliations:** 1 Wildlife Epidemiology Laboratory, College of Veterinary Medicine, University of Illinois, Urbana, IL 61802, USAadamovi2@illinois.edu (L.A.);; 2 Department of Veterinary Clinical Medicine, College of Veterinary Medicine, University of Illinois, Urbana, IL 61802, USA; 3 Department of Medicine and Epidemiology, University of California-Davis, Davis, CA 95616, USA

**Keywords:** bearded dragon, dermatomycosis, fungal, *Nannizziopsis guarroi*, *Pogona vitticeps*

## Abstract

*Nannizziopsis guarroi* causes dermatomycosis in lizards and snakes. Little is known about the environment’s role in transmission of the fungus. The environments of bearded dragons experimentally inoculated with *N. guarroi* were cultured weekly to assess the presence of viable *N. guarroi*. Four of six (4/6, 66.67%) enclosures demonstrated an environmental presence of *N. guarroi* prior to the observation of clinical lesions in the bearded dragon housed there. The environments were positive for *N. guarroi* growth 7–28 days prior to lesion development. The environment should be considered as a potential site of infection for naïve reptile hosts and environmental seeding may occur prior to the development of clinical nannizziomycosis in exposed lizards.

## Simple Summary

Bearded dragons are a common companion animal and are often infected by *Nannizziopsis guarroi*, a leading cause of dermatomycosis. In an experimental infection study, viable (culturable) *N. guarroi* were detected in the environment prior to the development of clinical signs of infection in most bearded dragons. These findings suggest that environment may play a role in disease transmission. Biosecurity measures should be used when handling bearded dragons to limit the spread of this fungus, even if the animal appears to be healthy.

## 1. Introduction

*Nannizziopsis guarroi* is the cause of “yellow fungus disease (YFD),” a colloquial term for dermatomycosis limited to reptiles [1,2,3]. In the most affected species, bearded dragons (*Pogona vitticeps*), the skin lesions may have a yellow coloration, although this term may be considered a misnomer as not all lesions are yellow in color [2,3]. While classically an infection of lizards, evidence of experimental cross-taxa infection to snakes exists [4]. Infected animals classically exhibit variably colored crusty or ulcerative cutaneous lesions that may progress to systemic mycosis [2,3,5,6]. Successful therapy is rarely reported [2] amongst the literature, which is replete with treatment failures [3,6,7].

While nannizziomycosis is considered contagious, transmission dynamics have yet to be elucidated [3,6,7,8]. Recent data indicate that environmental persistence may be an important factor, with *N. guarroi* reported to persist in water and on surfaces that reptiles under human care are likely to encounter in husbandry or clinical environments. [9]. In a recent infection trial assessing the performance of clinical diagnostics for *N. guarroi* detection, six adult bearded dragons were repeatedly exposed to *N. guarroi* [10]. Interestingly, despite dorsal application of conidial suspensions, most bearded dragons developed ventral cutaneous lesions, and no bearded dragons developed lesions at the sites of conidial application [10]. The current study aimed to assess if *N. guarroi* could be detected in the environment of infected beaded dragons. To this aim, we collected environmental samples from the enclosures of the bearded dragons experimentally infected in Wong et al., 2025 [10], to assess for the environmental presence of viable *N. guarroi* within this model. We hypothesized that viable *N. guarroi* would be detected in the environment prior to clinical lesion development. This article is a revised and expanded version of an abstract entitled “Viable *Nannizziopsis guarroi* can be detected from the Housing Environment of Experimentally Inoculated Bearded Dragons (*Pogona vitticeps*) Before Development of Clinical Lesions”, which was presented at the American Association of Zoo Veterinarians Annual Conference in 2024 [11].

## 2. Materials and Methods

Swabs of two environmental sites (newspaper substrate and basking rock) from the enclosures of singly housed lizards were sampled during an IACUC-approved (University of Illinois #22209) *N. guarroi* infection trial in six adult bearded dragons [10]. Lizards were housed and cared for as appropriate for the species [12]. The dorsa of each lizard were exposed to repeated cutaneous applications of molecularly confirmed *N. guarroi* conidial suspensions (2.59–3.71 × 10^7^ conidia/lizard, 7.09–11.2 × 10^4^ conidia/microliter). Latex gloves were worn and changed between the handling of each animal. Each lizard required 3 or 4 exposures until clinical manifestations of disease in the form of cutaneous lesions were noted (78–120 days post initial exposure) [10]. Additional exposures at increasing fungal burdens were required as the lizards did not develop clinical disease in the timeline that had previously been reported (15–31 days) [1].

**Figure 1:**
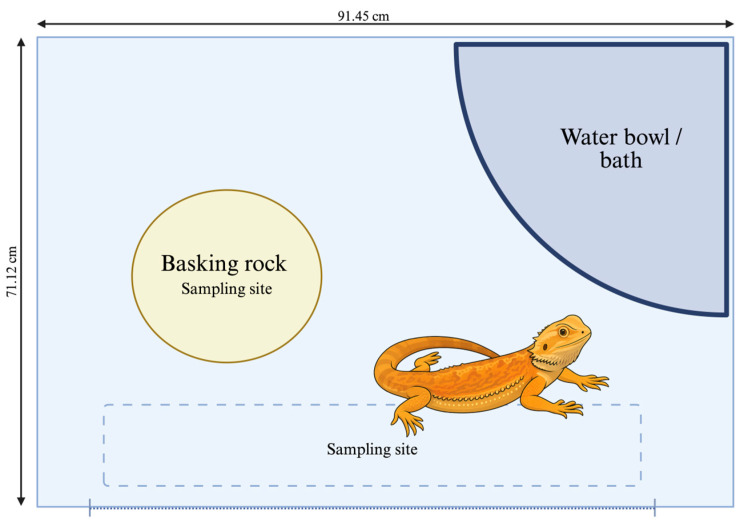
Enclosure floor plan of bearded dragons (*Pogona vitticeps*) experimentally infected with *Nannizziopsis guarroi*. The dotted line represents the front opening door and the window into the enclosure. The entire floor of the enclosure was covered in newspaper, with the area outlined with a dashed line representing a weekly sampling site (newspaper). The basking rock, maintained under the heat bulb, was the second sampling site. Created in BioRender. Keller, K. (2026) https://BioRender.com/wu4q0dw.

Each environmental site (newspaper substrate and basking rock) was swabbed once weekly for the first 14 weeks of the study course, spanning from day 0 prior to exposure through day 99 (Figure 1). Swabs were collected prior to weekly environmental cleaning that consisted of changing the newspaper substrate, removing dried debris, and cleaning the interior of the enclosure and the items in the enclosure (basking rock and water bowl) with dish soap and warm water. Newspaper was replaced in between sample times if visually contaminated with feces. Sample collection was performed prior to lizard fungal exposure events (days 0, 36, 64, and 99). The newspaper substrate surface in the front of the enclosure was chosen as it was outside of the basking area, representing a cooler temperature, while the rock was chosen as a sampling site as temperatures here were the highest temperatures expected in the enclosure. Both sites were frequently used by the lizards. Temperatures at each site were dynamic, as nighttime temps for both surfaces were ambient room temperature (23.3–24.4 °C, 74.0–76.0 °F), measured daily using a wall-mounted room thermometer. The highest temperatures during the day were 29.4–32.2 °C (85.0–90.0 °F) for newspaper and 35.0–37.8 °C (95.0–100.0 °F) for the basking rock, determined using laser thermometers, measured every 14 days.

Samples were collected with dry, sterile, cotton-tipped applicators; separate applicators were used for each site and clean gloves were placed prior to sample collection. Each surface was swabbed twenty times avoiding fecal and food material. Applicators were collected in sterile containers without media and within 2 h of collection samples were streaked onto single modified potato dextrose agar plates (Remel, Thermo Scientific, Lenexa, KS, USA) that included 50 mg gentamycin (Sigma-Aldrich, St. Louis, MO, USA), 400 mg cycloheximide (Acros Organics, Fair Lawn, NJ, USA), and 60,000 units/60 mg penicillin–streptomycin (Fisher Scientific, Waltham, MA, USA) for every one liter of agar. Agar plates were incubated at room temperature (21.1–22.2 °C 70.0–72.0 °F) for 13–14 days. Growth of *N. guarroi* was considered positive when colony morphology (white cottony growth) and direct microscopy findings (conidia and hyphae) consistent with published descriptions was noted [13,14], whether found in pure or mixed growth conditions. An environment was considered positive if either the sample from the rock or newspaper or both were positive for the growth of *N. guarroi*. Results were compared to the time for each lizard to develop clinically apparent disease [10]. The earliest DPIE for each sample site (newspaper and rock) was compared with a Mann–Whitney test and the proportion of samples that were positive for each sample site were compared using Fisher’s exact test. All statistical analysis was performed using commercial statistical software (GraphPad Prism 10.5.0 for Mac, Boston, MA, USA) with a significance of *p* < 0.05.

## 3. Results

Amongst the 204 cultures performed, representing two environmental samples from six enclosures over 17 sampling time points, 15 (7.4%) were positive for *N. guarroi* growth (Table 1). All the environmental samples were negative for growth prior to the initiation of the infection trial (day 0). Fifty cultures (24.8%) exhibited growth that was not consistent with the colony and/or microscopic morphology of *N. guarroi*. *N. guarroi* colony growth was white, round, elevated, and cottony, with yellow coloration noted on reverse agar. Hyphae seen under direct microscopy were often undulating and branching irregularly and fertile hyphae with sessile conidia were commonly seen. Free conidia were mostly cylindrical in shape and measured 3–6 µm in length.

Five of the six bearded dragons (animal IDs: B, C, E, F, J) developed clinical cutaneous lesions within the 14 weeks (99 days post initial exposure, DPIE) of this study’s period (Table 1) [10]. The five lizards that developed clinical lesions did so at DPIEs ranging from 78 to as late as 99, with a median time to clinical lesions of 99 days (mean 93.6 DPIE) [10]. One lizard (G) developed lesions after the cessation of environmental sampling at 120 DPIE [10]. In five of the six bearded dragons that developed cutaneous lesions, four (B, C, E, J) had at least one environmental sample that was positive for the growth of *N. guarroi* as early as 28 days prior to lesion development (a range of 7–28 days). Most environments were first positive for growth on 71 DPIE, with a single environmental sample being positive before that time (E, 64 DPIE).

Environmental samples from rock and newspaper were first positive at a median DPIE of 71 (with a range of 71–107 DPIE) and 71 DPIE (with a range of 64–107 DPIE), respectively. There was no difference in the time to first positive culture between sample sites (*p* = 0.64). Of the 102 cultures taken over 17 weeks from each sample site, 7 (3.43%) and 8 (3.92%) were positive for growth, taken from rock and newspaper, respectively. There was no difference in the proportion of positive culture results for each sample site (*p* > 0.99).

## 4. Discussion

Viable *N. guarroi* were cultured from most of the environments of experimentally infected bearded dragons prior to their development of clinical lesions. The lizards were exposed to *N. guarroi* outside of their enclosure in the form of conidial suspensions that were allowed to dry prior to their replacement in their enclosure. Thus, primary seeding of fungus from the conidial suspension directly to the enclosure is unlikely. It is possible that shed skin may have contaminated the environment. Furthermore, dried fungal elements may have become airborne and seeded the environment through vigorous movement, such as during prey item apprehension. Alternatively, cutaneous *N. guarroi* colonization may have preceded lesion development, allowing dissemination of the fungus to the environment via direct contact with colonized skin surfaces. Given that most bearded dragons had detection of DNA and/or viable *N. guarroi* from their skin prior to the development of lesions, this may be possible [10]. The samples collected from the bearded dragons used a whole-body swabbing technique, including both inoculation and non-inoculation areas, precluding the understanding of which body sites the fungi were present at [10]. However, most bearded dragons in the study developed ventral lesions despite dorsal exposure to the infectious agent [10], indicating that infection occurred either via contact of their ventral surfaces with a contaminated environment or via cutaneous colonization prior to development of disease at a distant site.

Our findings indicate the environment of a bearded dragon exposed to *N. guarroi* can become contaminated with viable *N. guarroi* and this has potential implications for clinical case management. A prior culture-based study has shown that surfaces including stainless steel, glass, or fabric found in typical bearded dragon husbandry structures or animal care facilities can support viable *N. guarroi* for up to 14 days and potentially longer [9]. This extended environmental persistence paired with our findings indicates the potential for an environmental role in disease transmission and a subsequent need for sound biosecurity protocols (quarantine and disinfection) to be used in breeding, veterinary, and pet store facilities to reduce transmission risk.

Two environmental sampling sites were selected within the bearded dragon enclosures that represented differences in thermal gradients, surface characteristics, and site-specific sanitation practices. *Nannizziopsis guarroi* is capable of growth across a broad temperature range (20–35 °C), which encompassed the temperatures recorded at both sampling sites [14]. The surfaces also differed in physical properties, ranging from the rough, low-porosity texture of the rocks to the smoother, more porous surface of the newspaper liner. Although both sites were subject to cleaning, sanitation methods differed: the rock surface was cleaned with soap and water weekly, whereas the newspaper was discarded weekly or more frequently, as it was inspected daily for contamination with feces. Despite the differences in sampling sites, no differences were observed in the proportion of culture-positive samples or in the time to first positive culture result. Future studies should evaluate more frequent sample times. A larger number of environments with concomitant enclosure use tracking may also bring valuable information on contamination dynamics. While viable *N. guarroi* were detected in the environment, this detection only occurred after several cutaneous exposures (DPIE 0, 36, and 64), when the first positive environmental sample was obtained. Interestingly, six of the seven cultures that were positive prior to the development of lesions occurred the week following an exposure event. Furthermore, 14 of the 15 positive cultures noted amongst the study timeline were positive only on the week following an exposure event. This clustering aligns with the timing of the large fungal burden being recently delivered (6–7 days prior) to the bearded dragon within that enclosure. Thus, it must be acknowledged that recent exposure was the largest factor in fungal environmental contamination in this experimental trial. The lack of serial positive environmental cultures is likely due to a combination of basic weekly sanitation efforts and, potentially, decreased cutaneous burden on the lizards, secondary to host immune activity.

While the results of this study have important clinical implications, there are also limitations to consider. Our study only included the environments of six bearded dragons and only included two substrates from each enclosure, with only once-weekly samples. It is unknown how these results translate to other environmental substrates and future studies should enroll a larger population and investigate more frequent sampling times to better understand contamination dynamics. Our findings only concerned the presence of fungus that was macroscopically and microscopically consistent with *N. guarroi* and we did not perform molecular confirmation of the isolates. While it is unlikely in this controlled research environment that other fungi (other *Nannizziopsis* spp., *Paranannizziopsis* spp., or *Ophidiomyces ophidiicola*) that share macroscopic and microscopic characteristics with *N. guarroi* were present, this cannot be confirmed. The minimum infective dose of *N. guarroi* and the host factors affecting susceptibility to disease development are not known. Future studies should consider introduction of a naïve host to a contaminated environment to test whether different environmental fungal burdens are sufficient to infect new hosts. Additional research into the pathogenesis of nannizziomycosis, particularly regarding the presence, extent, and duration of cutaneous colonization stages prior to lesion development, is also needed.

## 5. Conclusions

Paired with prior work showing prolonged environmental persistence, our findings suggest that the environment may play a role in the transmission of *N. guarroi*. While bearded dragons with dermatomycosis should be considered contagious, our findings suggest that bearded dragons infected with *N. guarroi* may contaminate the environment with viable *N. guarroi* and potentially infect naïve animals sharing the space without direct contact. Veterinarians, breeders, pet owners, and other animal care facilities should use effective disinfection protocols for infected or potentially infected lizards [15,16]. Future work should explore the role the environment plays in infection transmission in naturally infected lizards.

## Figures and Tables

**Table 1:** *Nannizziopsis guarroi* detection through fungal culture for six bearded dragon (*Pogona vitticeps*) (individual animal IDs: B, C, E, F, G, J) enclosures compared to time to lesion development (gray boxes) in an experimental *Nannizziopsis guarroi* (NG) infection model. DPIE = days post initial exposure, * = day of NG exposure for lizards that had not developed clinical lesions, + = positive for the growth of NG, − = negative for the growth of NG. Data for time to lesion development and days of inoculation sourced from previous materials [10].

	DPIE	0 *	8	15	22	29	36 *	43	50	58	64 *	71	78	85	93	99 *	107	113
B	Rock	−	−	−	−	−	−	−	−	−	−	+	−	−	−	−	−	−
	Newspaper	−	−	−	−	−	−	−	−	−	−	+	−	−	−	−	+	−
C	Rock	−	−	−	−	−	−	−	−	−	−	−	−	−	−	−	+	−
	Newspaper	−	−	−	−	−	−	−	−	−	−	+	−	−	−	−	+	−
E	Rock	−	−	−	−	−	−	−	−	−	−	+	−	−	−	−	+	−
	Newspaper	−	−	−	−	−	−	−	−	−	+	−	−	−	−	−	+	−
F	Rock	−	−	−	−	−	−	−	−	−	−	−	−	−	−	−	+	−
	Newspaper	−	−	−	−	−	−	−	−	−	−	−	−	−	−	−	+	−
G	Rock	−	−	−	−	−	−	−	−	−	−	−	−	−	−	−	−	−
	Newspaper	−	−	−	−	−	−	−	−	−	−	−	−	−	−	−	−	−
J	Rock	−	−	−	−	−	−	−	−	−	−	+	−	−	−	−	+	−
	Newspaper	−	−	−	−	−	−	−	−	−	−	+	−	−	−	−	−	−

## Data Availability

The original contributions presented in this study are included in the article. Further inquiries can be directed to the corresponding author.

## Abbreviation

The following abbreviation is used in this manuscript:

DPIE	Days post initial exposure
